# Basic research and clinical exploration of cold atmospheric plasma for skin wounds

**DOI:** 10.1002/btm2.10550

**Published:** 2023-05-26

**Authors:** Miaomiao Li, Jing Gao, Liyun Wang, Jia Liu, Chuyu Fu, Xingyu Yang, Shengquan Zhang, Xinwei Li, Shengyong Luo, Chunjun Yang

**Affiliations:** ^1^ Department of Dermatology and Venereology the Second Affiliated Hospital of Anhui Medical University Hefei Anhui China; ^2^ Anhui Provincial Institute of Translational Medicine Hefei Anhui China; ^3^ Department of Biochemistry and Molecular Biology, School of Basic Medical Sciences Anhui Medical University Hefei Anhui China; ^4^ Anhui Academy of Medical Sciences Hefei Anhui China

**Keywords:** basic research, clinical exploration, cold atmospheric plasma, wound healing

## Abstract

Skin wounds, such as burns, diabetic foot ulcers, pressure sores, and wounds formed after laser or surgical treatment, comprise a very high proportion of dermatological disorders. Wounds are treated in a variety of ways; however, some wounds are greatly resistant, resulting in delayed healing and an urgent need to introduce new alternatives. Our previous studies have shown that cold atmospheric plasma (CAP) has antibacterial activity and promotes cell proliferation, differentiation, and migration in vitro. To further advance the role of CAP in wound healing, we evaluated the safety and efficacy of CAP in vitro by irradiation of common refractory bacteria on the skin, irradiation of normal skin of rats and observing reactions, treatment of scald wounds in rats, and treating clinically common acute wounds. Our findings revealed that CAP can eliminate refractory skin bacteria in vitro; CAP positively affected wound healing in a rat scalding wound model; and direct CAP irradiation of low intensity and short duration did not lead to skin erythema or edema. CAP promises to be a new, economical, and safe means of wound treatment.

## Graphical abstract

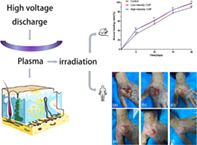

**Abbreviations**
APNTP: atmosphere pressure nonthermal plasma
CAP: cold atmospheric plasma
ESKAPE: *Enterococcus faecalis*, *Staphylococcus aureus*, *Klebsiella pneumoniae*, *Acinetobacter baumannii*, *Pseudomonas aeruginosa*, and *Enterobacter* spp.
MMPs: matrix metalloproteinases
PDGFR: platelet‐derived growth factor receptor

## 1 INTRODUCTION

As the body's largest organ and first line of defense, the skin performs multiple important functions, including providing a physical barrier against pathogens and performing absorption, secretion and excretion, thermoregulation, sensory, immune, respiratory, and endocrine functions, as well as participating in various functional activities of the entire body and maintaining an environment of stability within the body.^1^ Skin is highly susceptible to numerous factors that can lead to tissue damage and the onset of trauma. Acute wounds are very common in dermatology clinics, such as those resulting from trauma, burns, laser treatment, cryotherapy, or surgery. Most acute wounds heal quickly with early and aggressive intervention. However, some wounds become chronic due to delayed healing caused by local ischemia or wound infection, especially complicated infections.^2^ In addition to high morbidity and recurrence rates, wounds are often accompanied by high‐cost expenditures, which not only impose a considerable economic burden on patients, but also put tremendous pressure on national health care systems.

Wound repair is a complex series of distinct but partially overlapping processes, including phases of coagulation, inflammation, proliferation, and remodeling phases. There are multiple cellular interactions involved, including those between inflammatory cells, fibroblasts, keratinocytes, and endothelial cells, along with growth factors and enzymes.^3^, ^4^ Debridement, antiseptics, wound dressings, and negative pressure therapy are common ways of treating wounds. Newly developed nanomaterials have provided novel methods for wound treatment.^5^, ^6^, ^7^ However, painful treatment, repeated medical visits, and the great expenses involved in treatment lead to poor patient compliance. In addition, the emergence of antibiotic resistance further increases delays in the healing process.^8^, ^9^ Therefore, more effective treatment pathways are urgently needed. Exploring new skin trauma treatment methods, optimizing treatment protocols, and improving the effectiveness of trauma treatment are the main research directions for clinicians and researchers.

Plasma is a neutral, ionized gas that can be created under natural or artificial conditions. Due to its unique physical and chemical properties, it has applications in areas such as electrical, physical, chemical, energy, environmental, biological, medical, and materials science. Among them, cold atmospheric plasma (CAP) operates at temperatures similar to those of the human body (<40°C) and produces several active substances such as electrons, ions, neutral ions, UV light, heat, electric fields, etc. Based on the noncontact and painless natures, CAP is more suitable to deal with cells and human tissues.^10^ Global studies have shown that CAP has broad application prospects in the medical field. CAP not only shows positive effects in disinfection and sterilization,^11^, ^12^, ^13^, ^14^ beauty,^15^ cell proliferation,^16^ wound healing,^17^, ^18^, ^19^, ^20^, ^21^, ^22^, ^23^, ^24^, ^25^, ^26^, ^27^, ^28^, ^29^, ^30^ inhibiting tumor growth,^31^, ^32^, ^33^, ^34^ but also has therapeutic action on a variety of skin diseases (such as actinic keratosis,^35^ atopic dermatitis,^36^ pruritus,^37^ skin infectious diseases,^38^ psoriasis,^39^ disfiguring skin diseases,^40^ etc.). In addition, CAP can also be used as an efficient, environmentally friendly, and biocompatible cross‐linking agent. CAP‐treated biological dressings showed even better biological effects^41^, ^42^; and other new CAP treatment modalities, such as plasma knife and plasma cupping, are now also being developed.^43^, ^44^

The most widely used CAP application is the healing of different wound types. Researchers have found that CAP is essential for wound healing, but the specific cellular and molecular mechanisms by which it promotes tissue repair still need further investigation. In the present study, we investigated the antibacterial effect on the skin surface, the stimulatory response of CAP on local skin, and the therapeutic effect of CAP on clinical acute wounds. Based on the results of in vivo and ex vivo experiments, we concluded that CAP is expected to be a novel effective treatment modality for skin wounds.

## 2 RESULTS

### 2.1 CAP effectively kills common bacteria on refractory wounds

**FIGURE 1:**
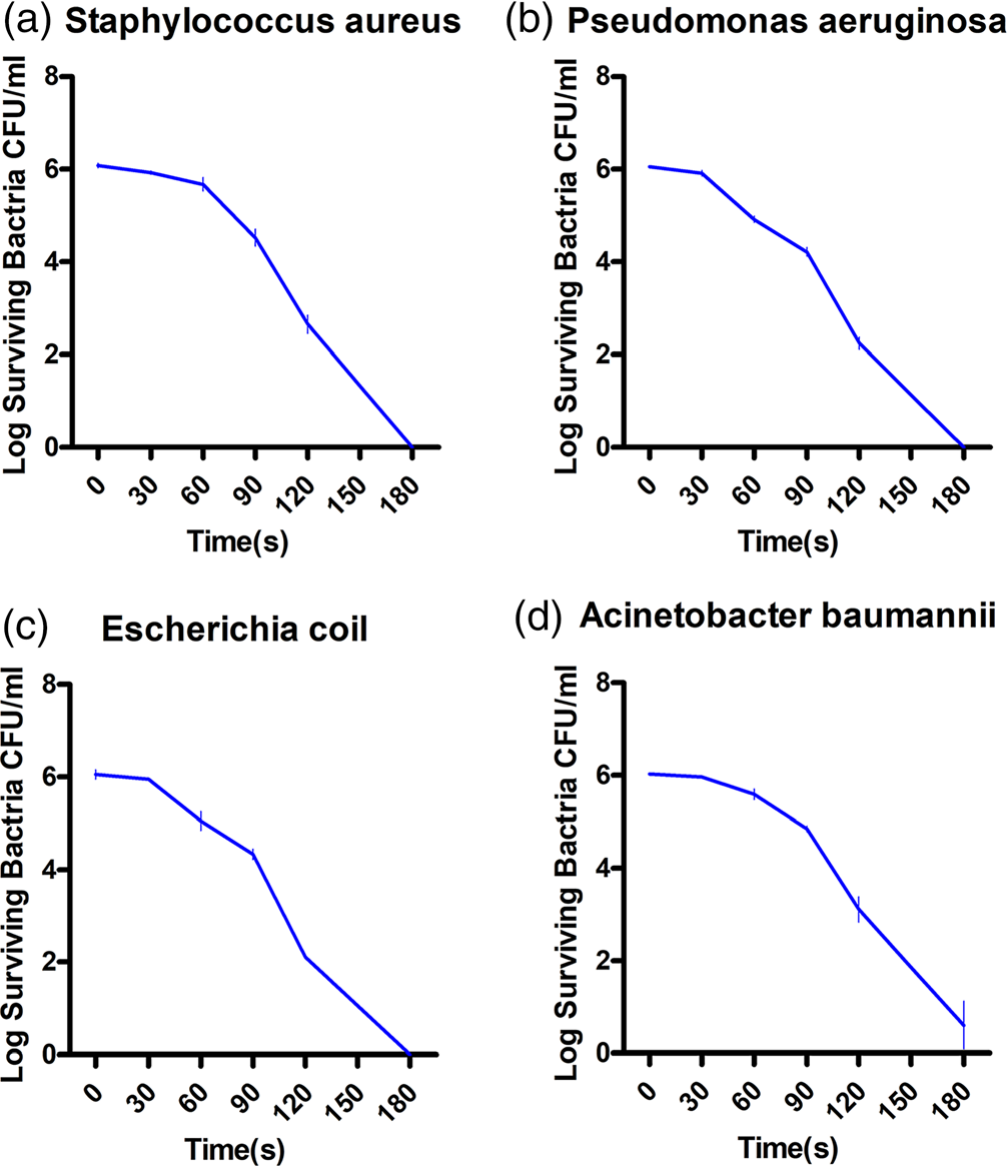
Changes in the number of viable colonies after 30, 60, 90, 120, and 180 s of exposure to CAP with four types of bacteria in refractory wounds. (a) *Staphylococcus aureus*, (b) *Pseudomonas aeruginosa*, (c) *Escherichia coli*, and (d) *Acinetobacter baumannii*.

Four common refractory wound bacteria (*Staphylococcus aureus*, *Pseudomonas aeruginosa*, *Escherichia coli*, and *Acinetobacter baumannii*) were treated with CAP in vitro. The number of surviving bacteria changed after irradiation at different times (30, 60, 90, 120 and 180 seconds) as shown in Figure 1 (the number of colonies counted by the plate counting method was converted into logarithmic values). After 24 h of bacterial culture, the initial bacterial count was 6.0 log 10 CFU/mL, and the number of bacteria gradually decreased with the extension of the CAP treatment. When using CAP treatment for 90 s, the number of surviving colonies was approximately 4.0 log 10 CFU/mL, while with 180 s of treatment, three bacterial species were almost killed, with the exception of *A. baumannii*. The results show that CAP has a sterilization effect in vitro.

### 2.2 Observation of CAP irradiation on local skin in rats

**FIGURE 2:**
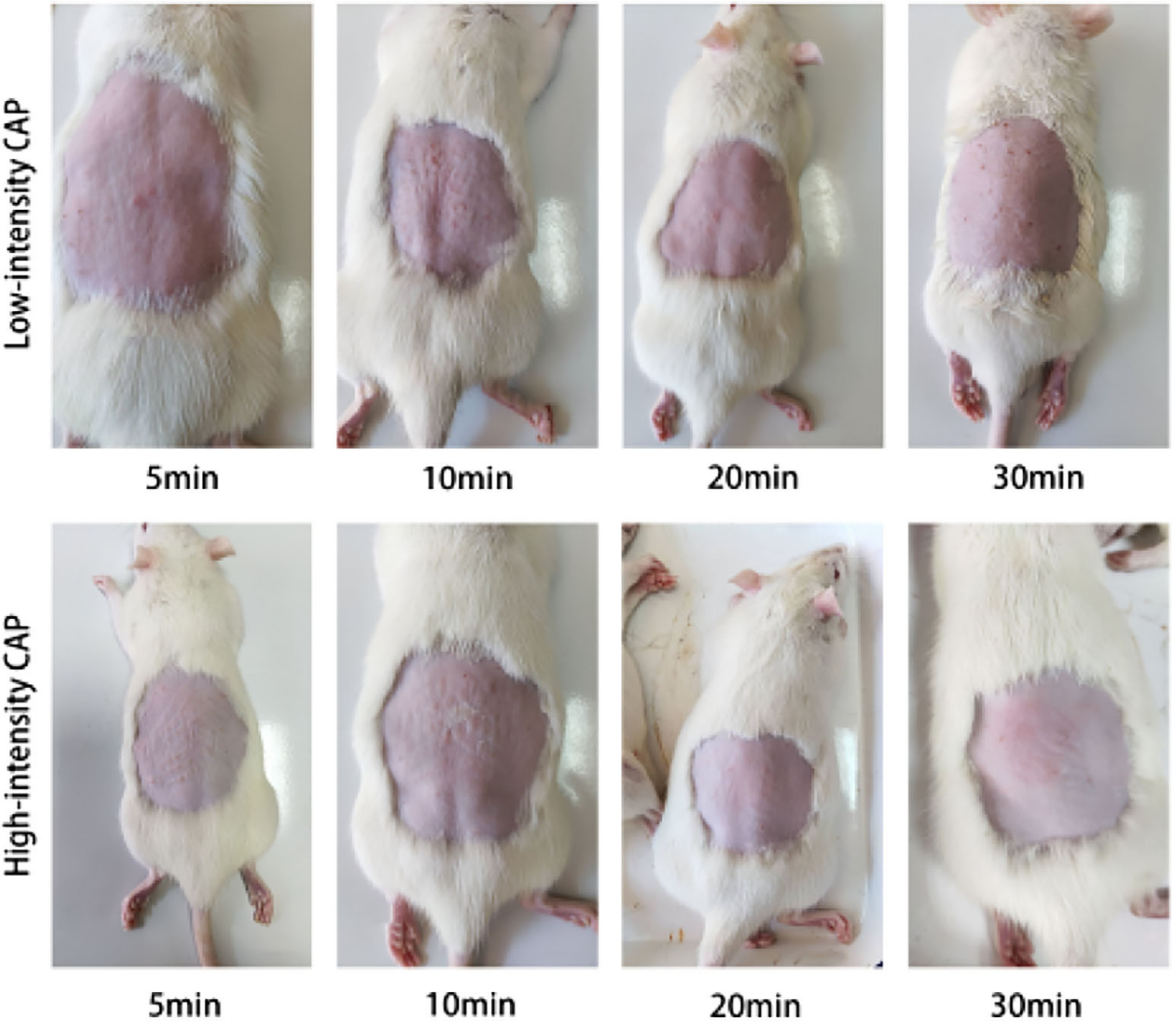
Local skin images of rats after receiving low‐intensity and high‐intensity CAP irradiation for 5, 10, 20, and 30 min, respectively. CAP, cold atmospheric plasma.

**FIGURE 3:**
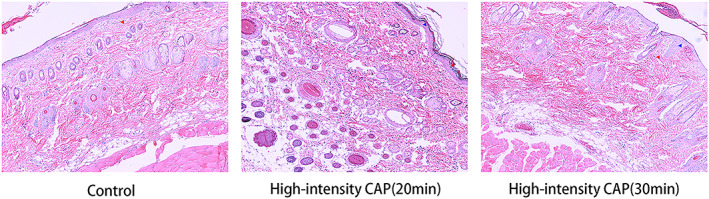
HE staining of erythematous or edematous skin tissues, with enlarged cell gaps indicated by blue arrows and inflammatory cells shown by red arrows. The magnification of these images is 100×. HE, hematoxylin–eosin.

We show the irritation scores of CAP irradiated rat skin in Tables 1 and 2, and images of localized skin irritation in rats by CAP in Figure 2, and show the skin histomorphometric results in Figure 3. In the high‐intensity CAP treatment group, when the irradiation time was 30 min, all three rats showed irritation reactions (erythema, edema, or both) on the dorsal skin, and the high‐intensity CAP irradiation of the skin for 30 min was assessed as moderately irritating. When the irradiation was continued for 20 min, one rat showed slight erythema on the skin of the experimental site, which was assessed as mildly irritating. No rats showed skin abnormalities at the experimental site when irradiated for 10 min or 5 min, which was assessed as nonirritating. In the low‐intensity CAP treatment group, no significant irritation was observed even after 30 min of continuous CAP skin irradiation. Histopathology showed a slight cutaneous edema after high intensity CAP treatment compared to the normal skin, while there was no significant difference in inflammatory cells.

**TABLE 1:** Skin irritation score scale for CAP‐irradiated rats.

	Score				Score		
	Erythema/edema				Erythema/edema		
Grouping	0 h	24 h	48 h	Grouping	0 h	24 h	48 h
High‐intensity 30 min	1/1	2/0	2/0	Low‐intensity 30 min	0/0	0/0	0/0
	0/1	1/0	1/0		0/0	0/0	0/0
	1/0	2/0	2/0		0/0	0/0	0/0
High‐intensity 20 min	0/0	0/0	0/0	Low‐intensity 20 min	0/0	0/0	0/0
	1/0	1/0	1/0		0/0	0/0	0/0
	0/0	0/0	0/0		0/0	0/0	0/0
High‐intensity 10 min	0/0	0/0	0/0	Low‐intensity 10 min	0/0	0/0	0/0
	0/0	0/0	0/0		0/0	0/0	0/0
	0/0	0/0	0/0		0/0	0/0	0/0
High‐intensity 5 min	0/0	0/0	0/0	Low‐intensity 5 min	0/0	0/0	0/0
	0/0	0/0	0/0		0/0	0/0	0/0
	0/0	0/0	0/0		0/0	0/0	0/0

Abbreviation: CAP, cold atmospheric plasma.

**TABLE 2:** Skin irritation index score in CAP‐irradiated rats.

	Score				Score		
Grouping	0 h	24 h	48 h	Grouping	0 h	24 h	48 h
High‐intensity 30 min	1.3	1.7	1.7	Low‐intensity 30 min	0	0	0
High‐intensity 20 min	0.3	0.3	0.3	Low‐intensity 20 min	0	0	0
High‐intensity 10 min	0	0	0	Low‐intensity 10 min	0	0	0
High‐intensity 5 min	0	0	0	Low‐intensity 5 min	0	0	0

Abbreviation: CAP, cold atmospheric plasma.

### 2.3 Efficacy of CAP in the treatment of the scalding model in rats

**FIGURE 4:**
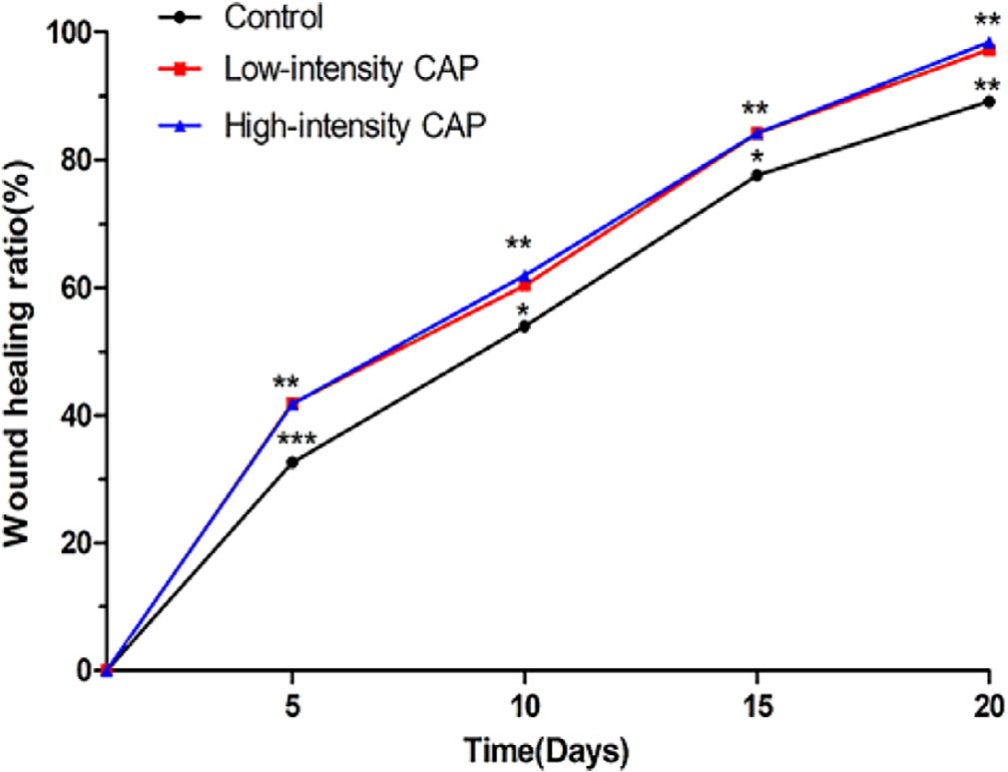
Folding graph of the wound healing rate over time in the control group, the low‐intensity CAP group, and the high‐intensity CAP group. CAP, cold atmospheric plasma.

**FIGURE 5:**
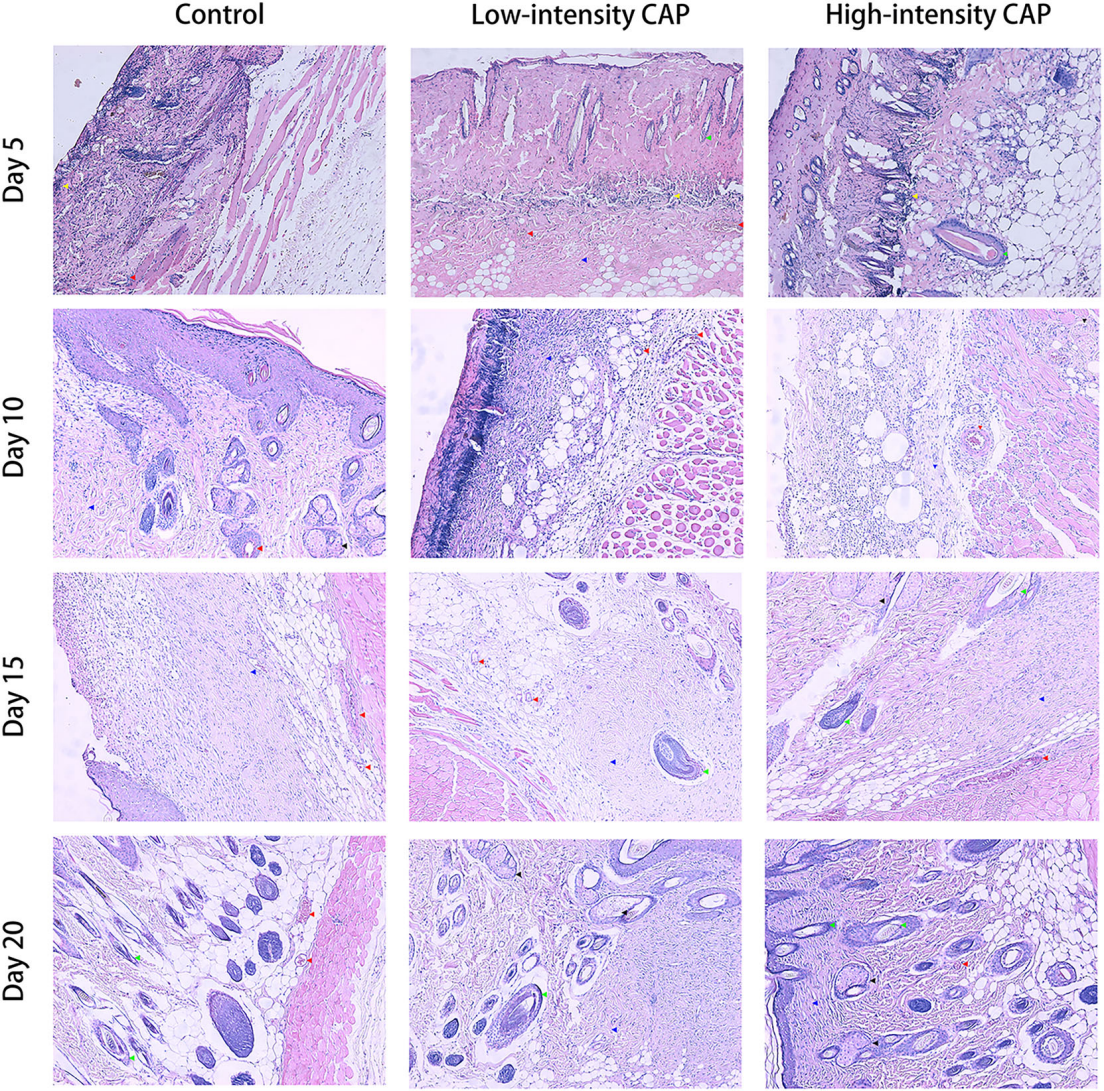
Representative histological features of rat skin: from top to bottom are the images of rat skin on day 5, 10, 15, and 20, respectively, with the control, low‐intensity CAP, and high‐intensity CAP groups, respectively in each row from left to right (yellow, red, green, black, and blue arrows point to inflammatory cells, neovascularization, hair follicles, sebaceous glands, and fibrous components, respectively). The magnification of these images is 100×. CAP, cold atmospheric plasma.

A scald wound model was constructed in rats. They were randomly grouped and treated with high‐ and low‐intensity CAP, and each rat was equipped with its own control. Statistics were performed on the 1st, 5th, 10th, 15th, and 20th days of treatment. It was found that the CAP group had faster wound healing compared to the control animals, and the difference was statistically significant (Figure 4, *p* < 0.05). This indicates that CAP treatment can promote the healing of scald wounds. Conversely, there was no difference in the rate of wound healing between the two CAP groups of different intensities. The histomorphological results showed that on day 5, inflammatory cell infiltration was visible in all groups, and neovascularization and skin accessory structures such as hair follicles and sebaceous glands were visible in varying degrees in the CAP treatment group but not in the control group; on day 10, neocytes and blood vessels were visible in the control group, but the skin structure hierarchy was disordered; in the CAP treatment group, epithelial cells were fully differentiated, with a clearly visible cell hierarchy. On day 15, the epidermal layer of the control group was clear, and the proliferated fibers were disorganized; in the CAP treatment group, the cell layer was clear, and the fibers were proliferated and neatly arranged; on day 20, the formation of blood vessels and epithelial differentiation of the traumatic surface were visible in all groups, and the physiological structure of the skin was restored (Figure 5).

### 2.4 Observation of CAP with the treatment of clinical acute wounds

CAP was used to treat six clinical acute wounds (one case of trauma formation after laser treatment, two cases of post‐freezing wounds, and three cases of postsurgical wounds). The patients' clinical data and treatment course are shown in Table 3. It was found that after 8–22 times of CAP treatment, all wounds were nearly cured. One patient had only three times of CAP treatment because of the distance to the place of residence, but the wound healed effectively. All patients who received treatment reported either no or tolerable pain during treatment. No skin irritation reactions such as erythema and edema were observed in all patients.

**TABLE 3:** Patient clinical data and treatment process.

Cases	Gender/age	Department/diagnosis	Previous treatment modalities	Times of CAP treatment	Results	Adverse reactions
1	Male/10 years old	Neck/scarring	Fractional CO_2_ laser	22	Cured	None
2	Female/73 years old	Hand/eczema	Glucocorticoid ointment, cryotherapy	9	Cured	None
3	Female/88 years old	Temporal/leather horn	Cryotherapy	8	Cured	None
4	Female/74 years old	Perioral/Basal Cell Carcinoma (BCC)	Surgical treatment	3	Effective	None
5	Female/77 years old	Temporal/Actinic Keratosis (AK)	Surgical treatment	10	Cured	None
6	Male/67 years old	Ankle/skin ulcers	Fibroblast growth factor gel, antibiotic ointment, red light exposure	14	Cured	None

Abbreviation: CAP, cold atmospheric plasma.

**FIGURE 6:**
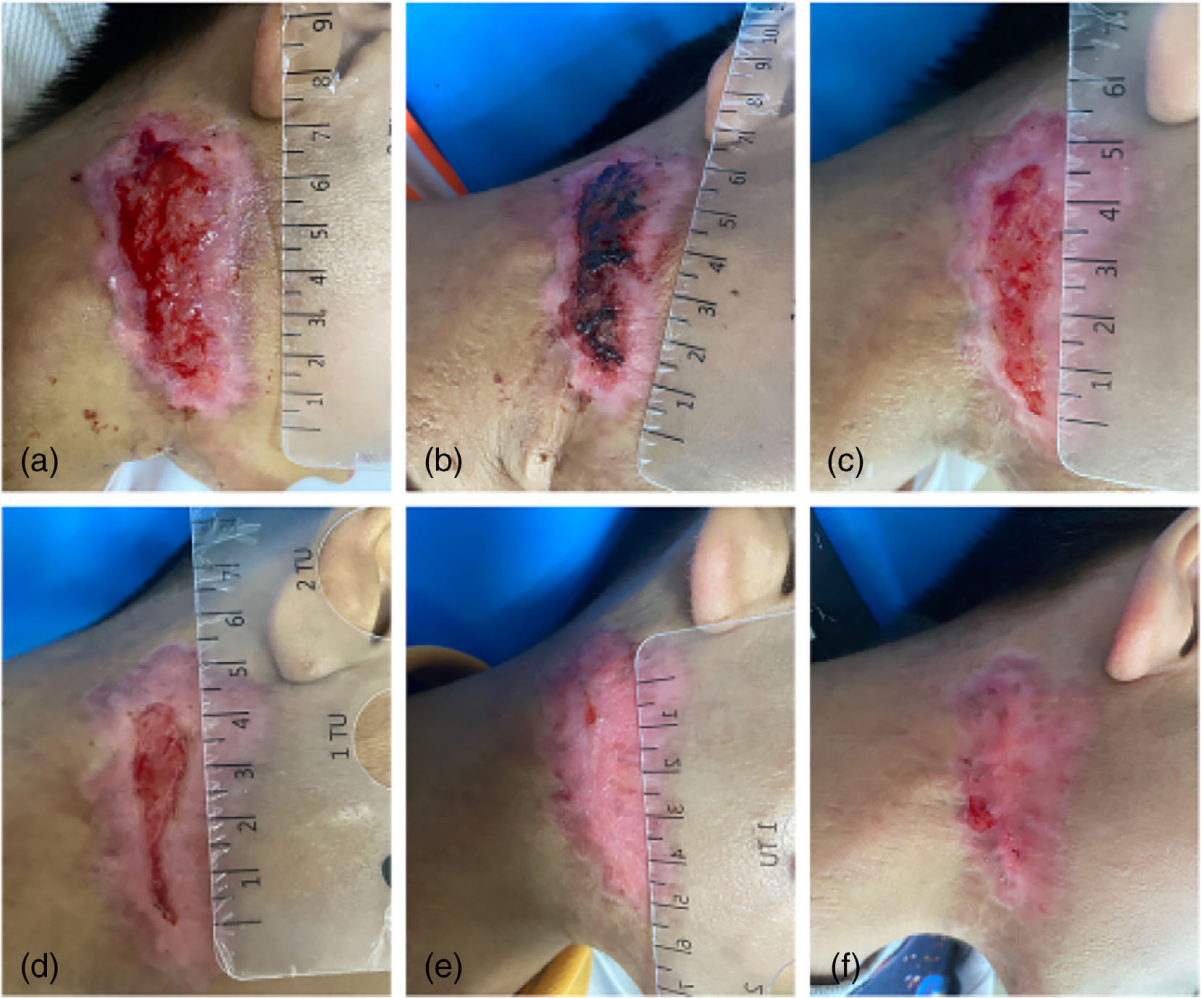
Images of wound healing before and after CAP treatment. (a) Before the treatment; after receiving (b) 1, (c) 7, (d) 12, (e) 18, and (f) 22 times of CAP treatment. CAP, cold atmospheric plasma.

*Case 1*: A 10‐year‐old male patient presented to our department with localized scar hyperplasia and underwent fractional CO_2_ laser treatment. However, after laser treatment, the wound developed breakdown and oozing. After full communication with the patient's family, CAP treatment was agreed upon. After four times of CAP treatment, the wound exudation decreased and some of the wounds crusted over. After 9 consecutive days of treatment, the wound had healed significantly. After a total of 22 times of CAP treatment, the wound was healed (Figure 6).

**FIGURE 7:**
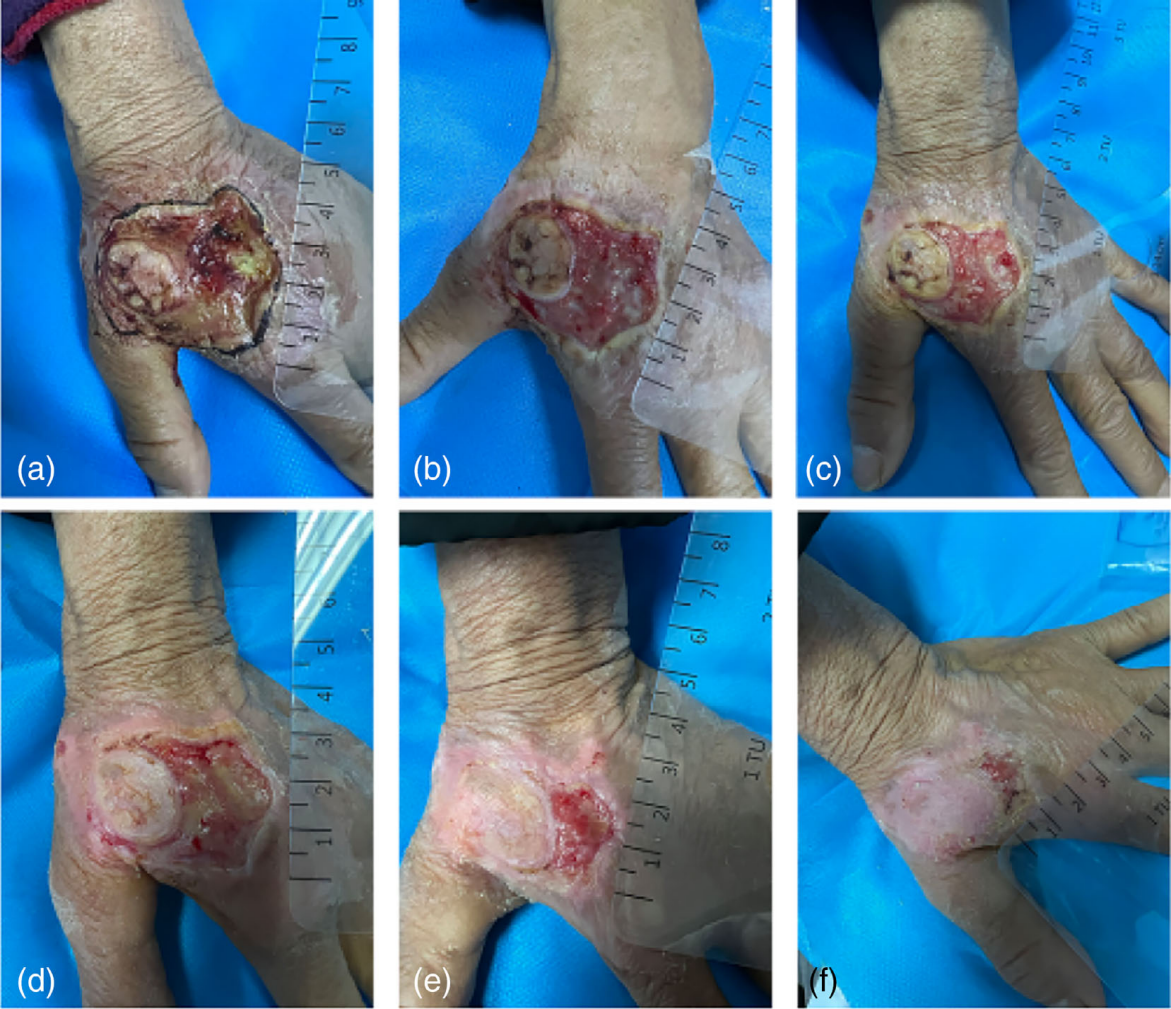
Images of wound healing before and after CAP treatment. (a) Before the treatment; after receiving (b) 2, (c) 4, (d) 5, (e) 8, and (f) 9 times of CAP treatment. CAP, cold atmospheric plasma.

*Case 2*: A 73‐year‐old woman was diagnosed with hand eczema and was treated with a combination of glucocorticoid ointment and cryotherapy, after which hand ulcers developed. After communication with the patient, CAP treatment was initiated. At the end of the second CAP treatment, wound exudation was significantly reduced, and the wound was completely healed after nine CAP treatments (Figure 7).

**FIGURE 8:**
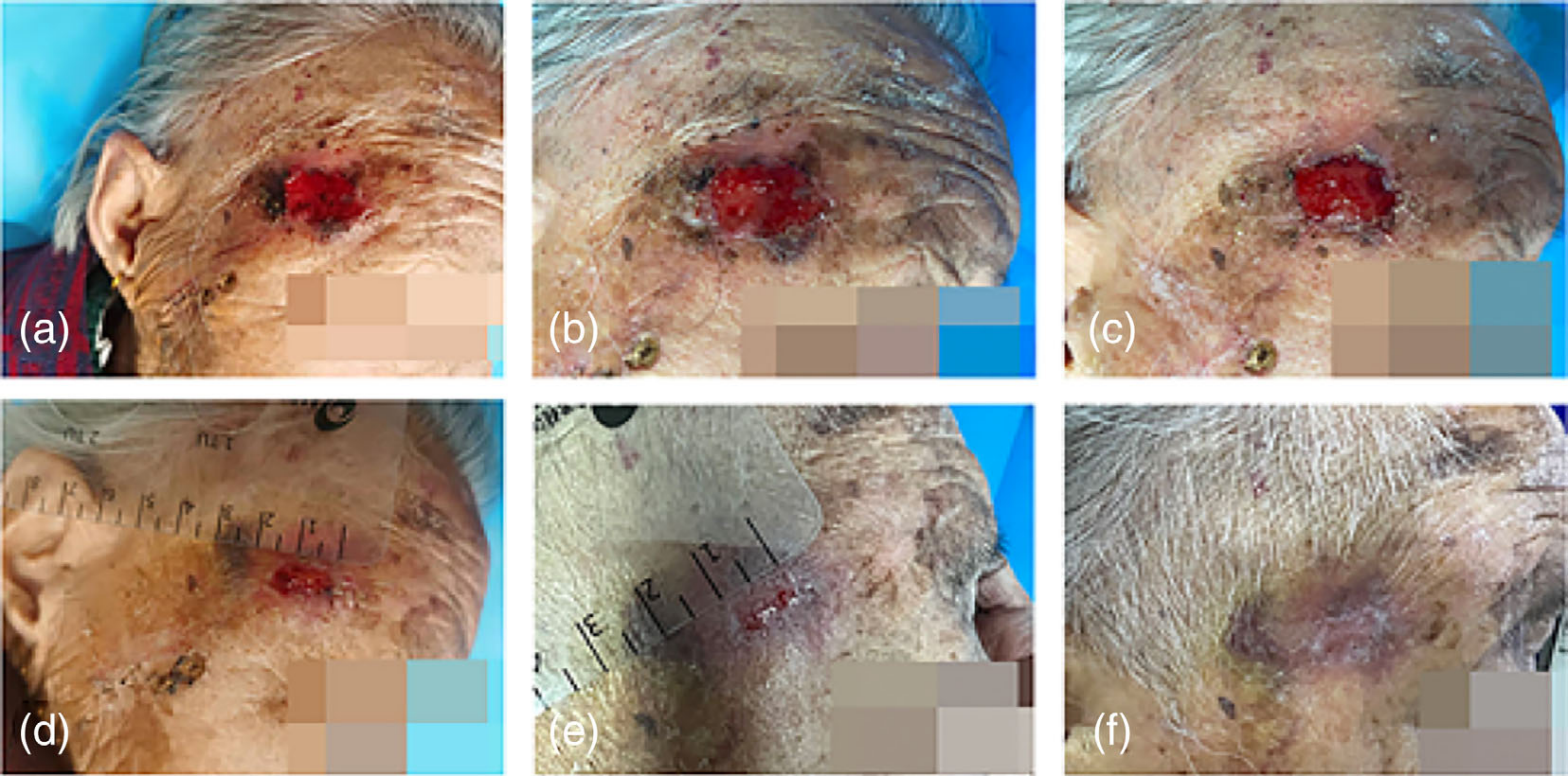
Images of wound healing before and after CAP treatment. (a) Before the treatment; after receiving (b) 2, (c) 3, (d) 5, (e) 7, and (f) 8 times of CAP treatment. CAP, cold atmospheric plasma.

*Case 3*: An 88‐year‐old woman developed a right temporal cutaneous horn. She declined surgical treatment and requested local cryotherapy; however, ulceration of the wound occurred after treatment. After communication with the patient, CAP treatment was performed, and the wound healed completely after eight times of CAP treatment (Figure 8).

**FIGURE 9:**
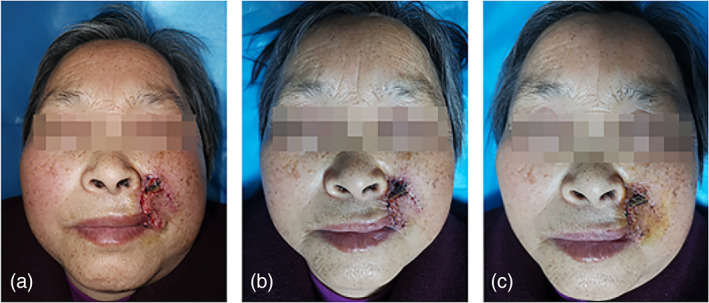
Images of wound healing before and after CAP treatment. (a) Before the treatment; after receiving (b) 1 and (c) 3 times of CAP treatment. CAP, cold atmospheric plasma.

*Case 4*: A 74‐year‐old woman was diagnosed with facial basal cell carcinoma and subsequently underwent an enlarged mass excision and adjacent metastatic flap repair. However, the wound healed poorly after surgery. After communication with the patient, CAP treatment was performed once a day, and the wound healed significantly after three times of consecutive CAP treatment (Figure 9).

**FIGURE 10:**
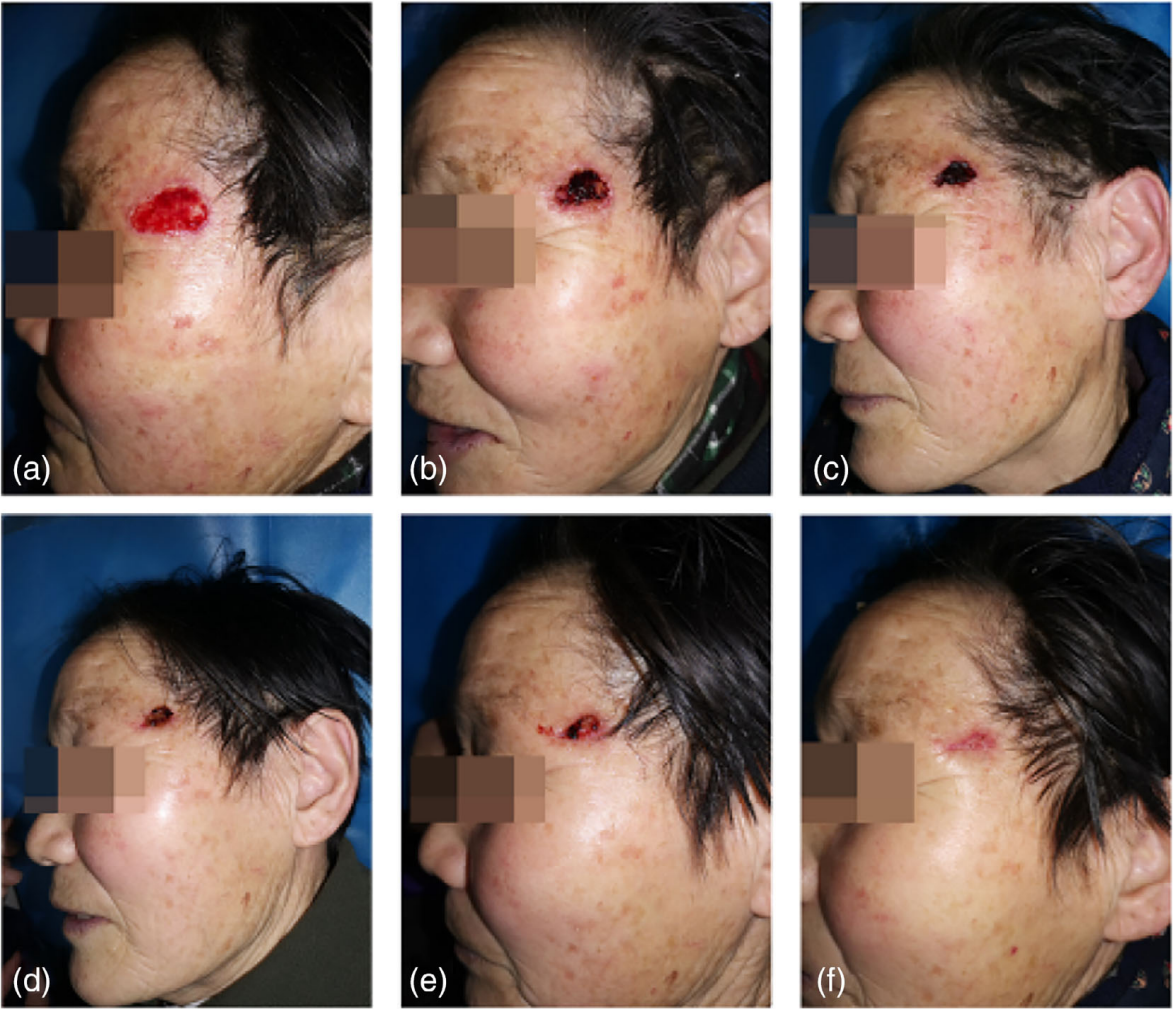
Images of wound healing before and after CAP treatment. (a) Before the treatment; after receiving (b) 1, (c) 3, (d) 5, (e) 7, and (f) 10 times of CAP treatment. CAP, cold atmospheric plasma.

*Case 5*: A 77‐year‐old woman was diagnosed with actinic keratosis of the left temporal region. The patient underwent surgical resection, but healing was not successful. After 10 times of CAP treatment, the wound healed (Figure 10).

**FIGURE 11:**
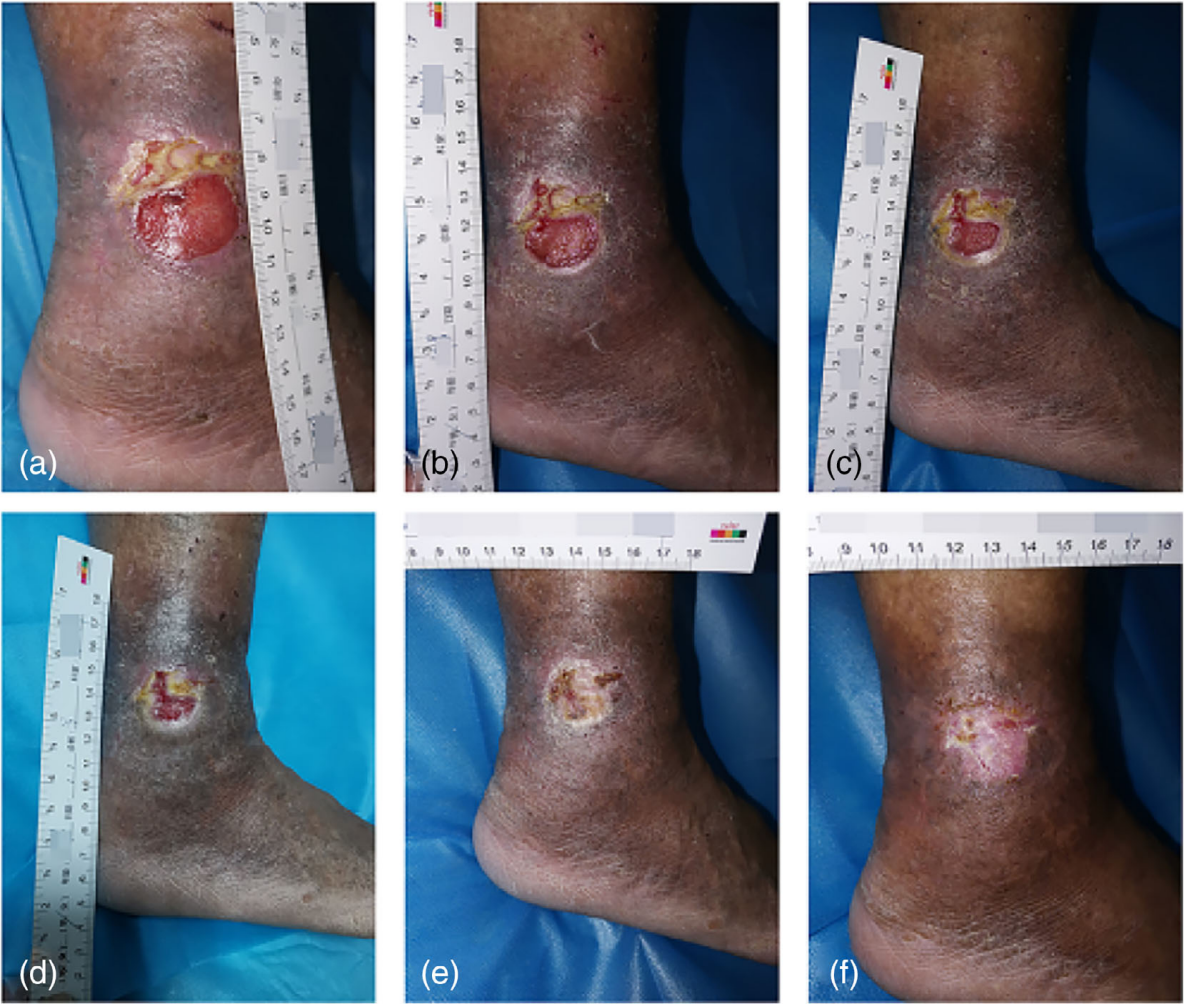
Images of wound healing before and after CAP treatment. (a) Before the treatment; after receiving (b) 3, (c) 6, (d) 8, (e) 11, and (f) 14 times of CAP treatment. CAP, cold atmospheric plasma.

*Case 6*: A 67‐year‐old male with poorly healed wounds after varicose veins in the lower extremities had been treated with fibroblast growth factor gel and antibiotic ointment combined with infrared irradiation, with no significant improvement of the ulcerated surface; the patient was managed with 14 times of CAP treatment and the wounds healed without significant scar growth (Figure 11).

## 3 DISCUSSION

Wound healing is a complex process, and different parts of the wound may show different stages of healing, making it difficult for the ideal synchrony to be achieved between the various parts of the wound. The “ESKAPE” (*Enterococcus faecalis*, *Staphylococcus aureus*, *Klebsiella pneumoniae*, *Acinetobacter baumannii*, *Pseudomonas aeruginosa*, and *Enterobacter* spp.) group was first introduced in 2008 to describe treatment‐resistant and life‐threatening Gram‐positive and Gram‐negative bacterial pathogens that are often insensitive to antibiotics and rapidly develop multiple drug resistance,^45^ leading to high morbidity and mortality and increasing the economic and psychological burden on patients.^46^ In addition, biofilm formation leads to more complex infections, with the Centers for Disease Control and Prevention estimating that 1.7 million hospital‐acquired infections are caused by biofilms in the United States each year; biofilms also account for approximately 80% of 44 microbial infections, making an important factor in the virulence of both acute and chronic infections.^47^, ^48^, ^49^ With the increase in drug‐resistant bacteria and infections caused by complex bacteria, the single use of antibiotics often fails to achieve the desired therapeutic effect.^50^, ^51^

In this study, the number of surviving colonies of *S. aureus*, *P. aeruginosa*, *E. coli*, and *A. baumannii* gradually decreased in vitro as the CAP irradiation time was prolonged. The initial colony count of all bacteria was approximately 6.0 log 10 CFU/mL, and when treatment lasted for 90 s, the surviving bacteria count was approximately 4.0 log 10 CFU/mL. Irradiation lasting 180 s killed all bacteria except *A. baumannii*. Therefore, it was concluded that CAP can inactivate bacteria in vitro. Flynn et al.^52^ examined the bactericidal properties of atmospheric pressure nonthermal plasma (APNTP) in biofilm and planktonic bacteria of the ESKAPE group. APNTP showed rapid antimicrobial activity against ESKAPE pathogens growing in planktonic form. Among the most sensitive bacteria to APNTP, *Enterobacter cloacae* showed complete clearance within 45 s. *P. aeruginosa* was also eradicated in 60 s using the APNTP method. Inactivation of *E. faecalis*, *K. pneumoniae*, and *A. baumannii* took place within 120 s, while *S. aureus* required 240 s. Within 360 s, all ESKAPE pathogens in biofilm form were completely eliminated except for drug‐resistant *A. baumannii*. According to these findings, CAP has positive sterilization capabilities and can be used to treat complex infections, such as those caused by ESKAPE pathogens and biofilms.

Burns are the most common type of acute trauma and are more prevalent in low socioeconomic level populations and less developed areas. When skin integrity is disrupted, the large amount of necrotic tissue and protein‐rich exudate at the damaged site provides the energy needed for microbial proliferation, leading to increased microbial susceptibility at the trauma site. In addition to infection, burns lead to a variety of other unavoidable complications, including scarring and impairment of limb function, requiring long‐term rehabilitation, reconstruction, and anti‐scar therapy.^53^ Moreover, it has been reported that burns are associated with an increased risk of cardiovascular disease, neurological disease, musculoskeletal disease, gastrointestinal disease, diabetes, infections, anxiety, depression, and tumor growth.^54^ In this study, we conducted a preliminary exploration of the effectiveness of air‐sourced CAP for treating scald wounds in rats, with daily CAP treatment for 3 min at a time for 20 days. Wound healing was found to be faster in the CAP group compared to the control group. The histomorphological rat skin findings showed that CAP treatment had a positive effect on the formation of blood vessels and granulation tissue and accelerated the re‐epithelialization process.

Similarly to our results, He et al.^17^ treated diabetic mice with helium, 90‐s CAP, or 180‐s CAP for 14 days and showed that CAP improved wound healing without a difference between exposure times. Lou et al.^26^ found that CAP treatment resulted in a significant reduction in wound area and a decrease in inflammatory cell infiltration compared to the control group. In another study, a single CAP treatment resulted in significantly faster wound healing, while CAP combined with negative pressure therapy and bone marrow mesenchymal transplantation further accelerated the healing process.^27^ These reports suggest a possible synergistic effect of CAP when combined with other therapies to promote wound healing. As a limitation of our study, we did not examine the mechanisms of CAP‐associated wound healing at a specific molecular or genetic level. Nonetheless, researchers have found that CAP enhances wound healing primarily by promoting angiogenesis, accelerated NO synthesis, and enhanced expression of PDGFR and CD31 pro‐angiogenic markers.^28^ Kisch et al.^29^ found that CAP can regulate tissular oxygen pressure and improve skin microcirculation, which is another key factor in wound healing and angiogenesis. Additionally, CAP promotes wound healing by regulating collagen deposition, reducing extracellular matrix degradation, and regulating wound healing stage‐dependent proteases [e.g., matrix metalloproteinases (MMPs)].^30^ In future studies, we will further explore the mechanisms by which CAP promotes wound healing.

Surgery, laser therapy, and cryotherapy are common clinical treatments in dermatology. With technological advances, dermatological surgery and dermatological aesthetics have gained unprecedented opportunities for development. However, the adverse effects of pain, infection, and delayed wound healing have never been completely avoided. In 2017, Hartwig et al.^55^ used CAP to treat difficult‐to‐heal wounds after head and neck surgery and achieved satisfactory results. In our study, using CAP for acute wounds formed after trauma, cryotherapy, laser treatment, or surgery, we found a positive effect of CAP on acute wounds, especially on the healing of complex acute wounds.

National and international research findings confirm the differential effect of gender on the trauma healing process. Female mice showed an advantage in the trauma healing process, which was associated with the upregulation of adenosine A_2A_ receptor‐mediated angiogenesis by 17β‐estradiol^56^; Routley et al.^57^ found that estrogen and progesterone drive wound repair, angiogenesis and remodeling through activation of macrophages. Furthermore, Gilliver et al.^58^ showed increased type I collagen and fibronectin deposition in the wounds of male denuded rats compared to normal male rats, accompanied by a significant decrease in the activity of collagen digesting enzymes (MMP‐2 and MMP‐9). In general, estrogen was identified as a repair promoter that accelerates healing, whereas androgens are an inhibitor that delays injury recovery. In addition to sex hormones, the influence of gender on wound healing is manifested in other factors that are so complex that male and female organisms respond to injury repair, which is itself different. In another study by Gilliver et al.,^59^ they showed the differences in the area of unhealed wounds in ovariectomized females and male denuded animals, which may be related to a combination of macrophage migration inhibitory factor as well as unknown factors. In preclinical studies, to observe the outcome of CAP on wound repair, we used male Sprague Dawley (SD) rats to avoid the effect of sex differences on wound healing. As far as the results of the current study are concerned, CAP seems to have a consistent effect with estrogen on wound healing, and it is equally beneficial in accelerating wound healing. However, few animal experiments on the effect of CAP on wound healing have been observed with female animals as the study subjects. Whether CAP has an antagonistic effect with estrogen and thus has a negative effect on wound healing is currently lacking, and we will further improve this part in future studies.

Multiple studies have confirmed the ability of CAP to induce mutations, apoptosis, and DNA damage in the treatment of neoplastic diseases, but whether CAP is harmful to humans, cells, or tissues deserves more attention. Maisch et al.^60^ treated keratinocytes and fibroblasts with CAP for 2 min and no relevant toxic effects were observed. After repeated treatment with CAP for 2–10 min every 24 h for 5 days, no mutagenicity was detected in a mammalian cell gene mutation assay, while UV‐C exposure of cells induced DNA damage and mutagenesis. Schmidt et al.^61^ performed the first long‐term analysis of a mouse model (1 year of continuous follow‐up after completion of CAP treatment) and described the CAP‐treated traumatic areas without abnormal morphological changes histologically, no excessive scarring, or chronic inflammatory response; histology and radiology (magnetic resonance imaging) showed no malignant tumorigenesis in skin tissue or solid organs. Evert et al.^62^ also found that repeated CAP exposure did not lead to noninvasive lesions or squamous cell carcinomas in the oral mucosa of mice.

Different intensities of CAP were used to irradiate the normal dorsal skin for different time periods, and it was found that local skin erythema and edema appeared with increasing treatment time and intensity. At high intensity, the treatment time of 10 min was safe, and skin biopsy of the area where erythema and edema appeared revealed slight skin keratinization and edema, with no significant differences in inflammatory cells; with low‐intensity CAP, no skin irritation reaction was observed even after 30 min of continuous irradiation. After repeated validation, no significant adverse effects were found with the plasma devices used in the clinic and laboratory. However, CAP exhibits different electrical properties in different models (in vitro culture dishes, animal models, and for biomedical applications). How to compensate for these differences considering the currently available in vitro and ex vivo evidence is critical to standardize treatment modalities for the safe application of CAP in biomedicine and to reduce any potential risks.^63^

Plasma medicine is continuing to evolve rapidly, and increasingly more studies are providing new evidence for the use of CAP as an alternative treatment for a variety of skin trauma and skin diseases. CAP is sensitive not only towards postsurgical wounds, laser, and cryotherapy wounds, but can also be used in the treatment of a wide range of skin diseases. This study confirms the potential application of CAP in wound healing, and CAP is expected to become a novel, economical, and safe treatment modality. However, the study also had the following limitations: (1) the CAP device used in this study only confirmed its efficacy and safety in trauma treatment through in vitro bactericidal effects and studies in rat scald models, and lacks more in‐depth mechanistic studies; (2) only male rats were observed for the study in the animal experiments, and whether there are gender differences in the effect of CAP on wound healing needs to be further explored; (3) no further studies on microbial infection of trauma and posttreatment conditions were conducted in the clinical study; (4) in the clinical study, patients had received various other treatments before CAP treatment, which may affect the final result; (5) the clinical sample of this study was very limited. To further confirm the efficacy and safety of CAP treatment, additional clinical samples should be included, randomized controlled observational studies should be conducted, and the treatment parameters should be explored.

## 4 CONCLUSIONS

CAP has an in vitro sterilizing effect on common skin trauma‐refractory bacteria (*S. aureus*, *E. coli*, *P. aeruginosa*, *A. baumannii*). Low intensity and brief CAP irradiation of normal skin does not lead to reactions such as erythema or edema, but high intensity and prolonged irradiation shows erythema and edema irritation reactions, and the probability and severity of irritation reactions increase with treatment time. CAP can promote the healing of local scald wounds in rats. Regarding the treatment effect of CAP on clinical common skin wounds, patients have high acceptance and mild adverse reactions, which has a positive impact on shortening the course of treatment. However, there is no accurate standard for the therapeutic dose of CAP for different types of wounds. Accurately controlling the therapeutic dose also remains a challenge which needs to be further explored and discovered in the future.

## 5 METHODS

### 5.1 Plasma equipment

**FIGURE 12:**
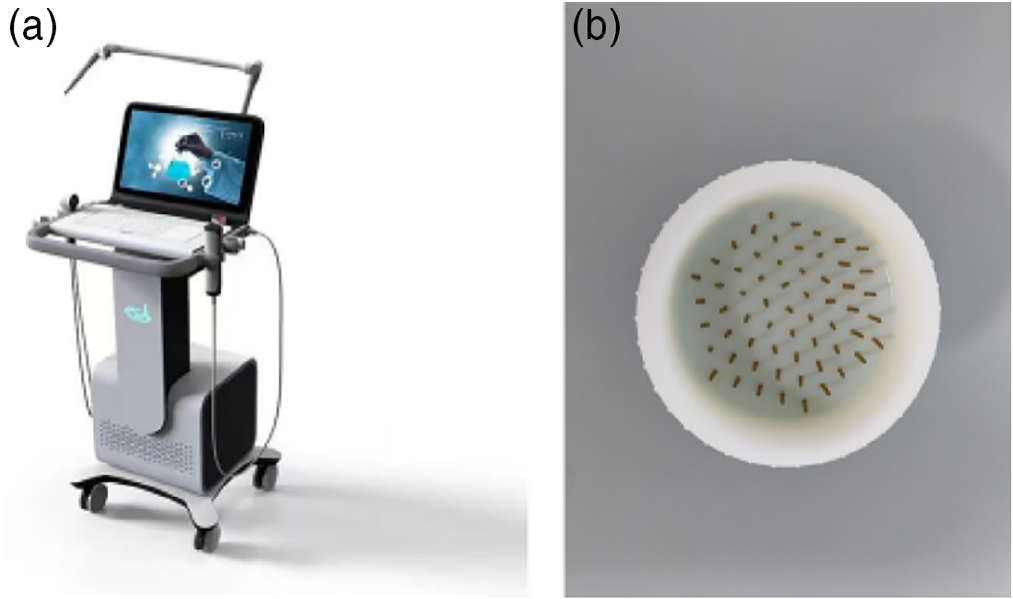
(a) The air‐sourced cold atmospheric plasma device. (b) High voltage discharge tip.

**FIGURE 13:**
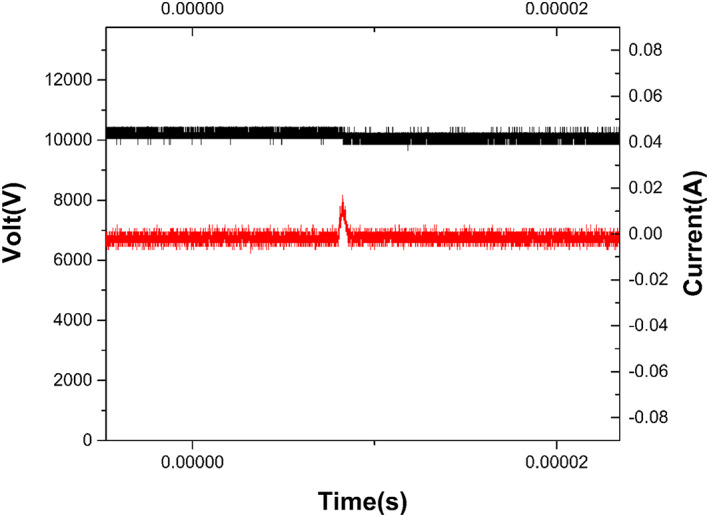
Current and voltage signal diagram of the plasma center point under specific parameters.

**FIGURE 14:**
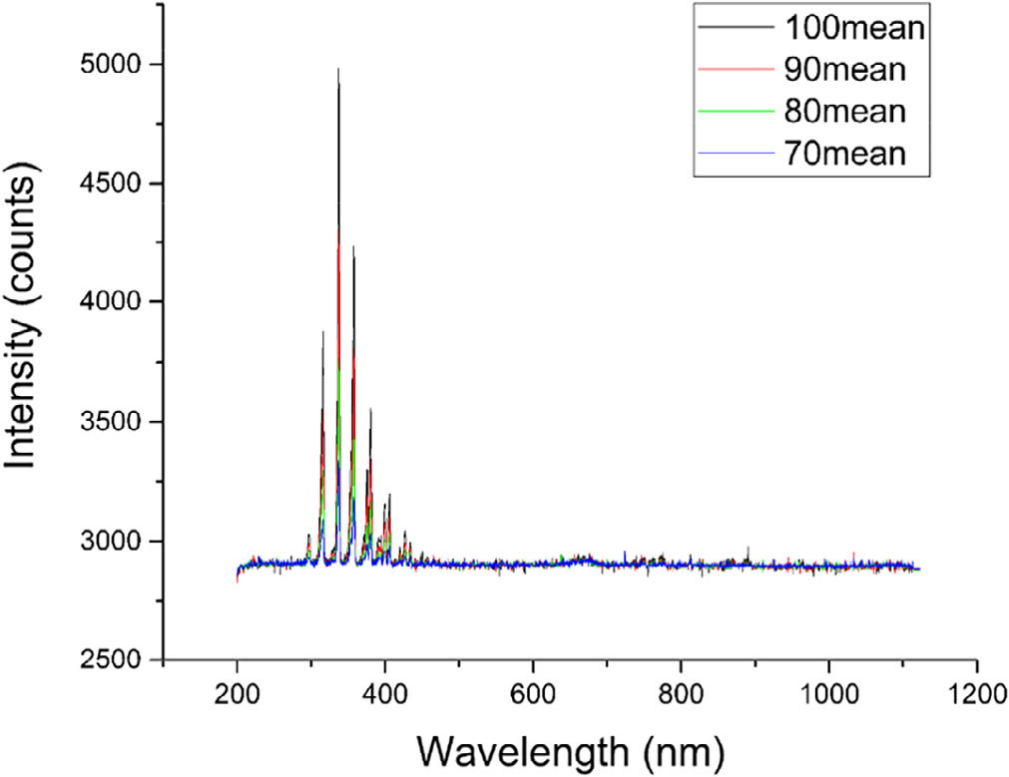
Emission spectra of plasma instruments.

The ST‐P101 plasma skin therapy instrument (Hefei CAS Ion Medical and Technology Devices Co., Ltd, Hefei, China) was used in this study (Figure 12). High voltage electrodes (copper needles with a tip radius of approximately 1 mm) are required, and a handheld electrode sheet is connected to the experimental animal limb, forming a closed loop. Plasma is generated through a high voltage electrode discharge with an adjustable voltage control range of 9–15 kV and a ballast resistance of 10 MΩ. During the experiment, the distance between the skin surface of the treated area and the multielectrode tip was kept at 10 mm, perpendicular to the skin surface. The plasma discharge voltage and current were monitored using an MSO 5104 digital oscilloscope equipped with a high voltage probe (P6015A) and a current probe (Tektronix P6021, Tektronix, Bracknell, United Kingdom); the current and voltage of the monitored plasma are shown in Figure 13. The emission spectra were recorded using an AvaSpec‐2048 spectrometer (Avantes, Apeldoorn, The Netherlands); the emission spectra of the plasma are shown in Figure 14.

### 5.2 Inactivation of common bacteria in refractory wounds by plasma

Tryptone, yeast extract, sodium chloride, and agar were added to deionized water in a concentration ratio of 10, 5, 10, and 20 g/L, respectively, sterilized at 121°C for 30 min, poured onto a 60‐mm sterile flat plate, left to cool and solidify, and then prepared for use. Further, 2 mL of bacterial suspension in 35‐mm sterile petri dishes was irradiated with CAP for 0, 30, 90, 120, and 180 s, respectively. Using a micropipette, 100 μL of diluted bacterial solution were added to the Lysogeny broth solid medium, spread evenly with a sterile coating rod, and then placed into a constant temperature incubator at 37°C for 24 h. The total number of surviving bacteria was obtained by counting the number of surviving bacterial colonies in the Petri dishes multiplied by the dilution factor.

### 5.3 Observation of the effect of plasma irritant on local skin in rats

SD rats (SPF grade, 180–220 g, males) were purchased from Zhejiang Viton Lihua Laboratory Animal Technology Company and housed in a standard breeding environment. Twenty‐four SD rats were numbered from 1 to 24 according to the body weight, and the random numbers were obtained using Excel software, so that each animal was coded with a different random number. The animals were reordered by “increasing” random numbers, and then divided into eight groups in sequential order with three replicates in each group. CAP irradiation was performed according to the treatment intensities and treatment durations specified in Table 4. The use of research animals in this study was reviewed and approved by the Laboratory Animal Welfare and Medical Ethics Committee of Anhui Medical University (No. LLSC20221118).

**TABLE 4:** Grouping of local skin irritation of CAP in rats.

Group	Treatment modality	Treatment intensity	Duration of treatment (min)
A1	CAP treatment	High‐intensity	5
B1			10
C1			20
D1			30
A2		Low‐intensity	5
B2			10
C2			20
D2			30

Abbreviation: CAP, cold atmospheric plasma.

At the start of the experiment, the weight of each rat was measured using an electronic scale. Each rat was anesthetized intraperitoneally using 40–50 mg/kg of 2% pentobarbital sodium. After successful anesthesia, the back of the rat was dehaired. The intensity of the CAP device was set at 80% (energy output 0.8 J/s) and the electrode piece was suspended at 1 cm from the surface of the rat's skin. Twelve adult rats were irradiated for 30, 20, 10, or 5 min; irradiation was repeated for the three rats in each group. The above treatment step was repeated at 60% (energy output 0.4 J/s). The skin surface damage (erythema or edema) of rats in each group was observed immediately after treatment, 24 h after treatment, and 48 h after treatment. The severity of erythema and edema was scored according to Table 5 (skin irritation response scoring criteria), and the skin irritation index was calculated by averaging the accumulated irritation scores of all animals in each group. The skin irritation intensity after CAP irradiation was assessed according to Table 6 (skin irritation intensity grading). After the observation, the skin tissues of rats with skin irritation reactions were fixed using 4% paraformaldehyde fixation, stained with hematoxylin–eosin (HE), and examined histomorphologically. The pathological tissue samples were stained and fixed by the Wuhan Safeway Biotechnology Company.

**TABLE 5:** Skin irritation reaction scoring criteria.

Skin irritation reaction	Severity grading	Score
Erythema	No erythema formation	0
	Barely visible	1
	Clearly visible	2
	Crust formation	3
Edema	No edema formation	0
	Barely visible	1
	Clearly visible	2
	Skin breakout	3

**TABLE 6:** Skin irritation intensity grading.

Skin irritation index	Stimulus intensity grading
0	Non‐irritating
0~	Mildly irritating
1.0~	Moderately irritating
3.0 ~ 6.0	Strongly stimulating

### 5.4 Observation on the efficacy of plasma treatment for the scalding model in rats

Forty SD rats were dehaired 24 h before the experiment. To establish the scald model, a metal cylinder with a base diameter of 15 mm was heated at a constant temperature of 85°C in water for 15 min. Next, the metal was placed on the left and right sides of the dehaired back of the rats for 5 s without pressure, and each rat had two identical traumas on the dorsum. Forty rats were sorted by body weight, and two rats with approximately the same body weight were paired and numbered 1 and 2, respectively. Twenty numbers were copied from the random number table. For odd numbers, the animal numbered 1 in the pairing group entered the high‐intensity CAP group and the animal numbered 2 entered the low‐intensity CAP group, and for even numbers, the opposite is true, so that 40 rats were randomly divided into two groups. The left side of each rat was coated with saline as the control group, and the right side was irradiated with high‐intensity and low‐intensity CAP according to the group. The irradiation method was the same as that of CAP irradiation in the study of rat skin safety and CAP treatment was performed once a day for 3 min at a time for 20 days. The area of dorsal trauma was recorded on treatment days 1, 5, 10, 15, and 20, and the dorsal skin was taken for HE staining to observe the histomorphological changes. All procedures were done by the same experimenter to avoid additional errors caused by intersubject variation.

### 5.5 Observation of plasma treatment with clinical acute wounds

From December 2021 to February 2022, six patients with skin wounds that did not respond well to conventional treatment protocols were selected from our outpatient clinic and wards, and CAP treatment was administered after communication with patients and their guardians, obtaining written consent. Pregnant and lactating women, people with dementia, and those who asked to be withdrawn halfway through were excluded from the study. This study was ethically reviewed by the Ethics Committee of the Second Affiliated Hospital of Anhui Medical University (No. YX2021‐029 (F1)).

Patients were placed in an appropriate position for full exposure of the treatment area and the lesions were cleaned with saline to remove foreign bodies. The traumas are treated with the ST‐P101 plasma skin treatment instrument. Different high voltage output electrodes are selected depending on the size of the lesion and the frequency of treatment depends on the severity of the wound (once a day or every other day) until the wound cured or nearly cured. A 30‐min observation was undertaken after each treatment. Three indicators were used to evaluate treatment effectiveness: cured (disappearance of clinical symptoms and signs), effective (improvement of clinical symptoms and signs), and ineffective (neither improvement nor aggravation of clinical symptoms and signs). The presence of adverse reactions (itching, pain, erythema, or edema at the irradiated area) was used to evaluate treatment safety for the human body. The skin lesions were photographed before and after each treatment.

### 5.6 Statistical analysis

Data were subjected to *t*‐tests and nonparametric tests using IBM SPSS Statistics 26 (IBM Corp. Armonk, NY, USA) to assess differences between experimental groups. Results were considered statistically significant at *p* < 0.05.

## AUTHOR CONTRIBUTIONS

**Miaomiao Li:** Data curation (equal); investigation (equal); methodology (equal); software (equal); writing – original draft (equal); writing – review and editing (equal). **Jing Gao:** Conceptualization (equal); data curation (equal); funding acquisition (equal); methodology (equal); software (equal); writing – original draft (equal); writing – review and editing (equal). **Liyun Wang:** Funding acquisition (equal); methodology (equal); writing – review and editing (equal). **Jia Liu:** Methodology (equal); writing – review and editing (equal). **Chuyu Fu:** Methodology (equal); writing – review and editing (equal). **Xingyu Yang:** Methodology (equal); writing – review and editing (equal). **Shengquan Zhang:** Conceptualization (equal); funding acquisition (equal); supervision (equal); writing – review and editing (equal). **Xinwei Li:** Methodology (equal); writing – review and editing (equal). **Shengyong Luo:** Methodology (equal); writing – review and editing (equal). **Chunjun Yang:** Conceptualization (equal); funding acquisition (equal); project administration (equal); supervision (equal); writing – review and editing (equal).

## FUNDING INFORMATION

This study was supported and funded by Natural Science Research Project of Anhui Colleges and Universities, Grant/Award number: KJ2020ZD19; Incubation Program of National Natural Science Foundation of China, The Second Affiliated Hospital of Anhui Medical University, Grant/Award number: 2021GQFY03; Research Fund of Anhui Provincial Institute of Translational Medicine, Grant/Award number: 2021ZHYX‐C48; Clinical Training Program of the Second Affiliated Hospital of Anhui Medical University, Grant/Award number: 2020LCZD21; Basic and Clinical Cooperative Research Promotion Program of Anhui Medical University, Grant/Award number: 2020XKJT042; and Research Fund of Anhui Medical University, Grant/Award number: 2022XKJ048.

## CONFLICT OF INTEREST STATEMENT

The authors declare no conflicts of interest.

### PEER REVIEW

The peer review history for this article is available at https://www.webofscience.com/api/gateway/wos/peer-review/10.1002/btm2.10550.

## ETHICS STATEMENT

The Laboratory Animal Welfare and Medical Ethics Committee of Anhui Medical University (No. LLSC20221118); The Ethics Committee of the Second Affiliated Hospital of Anhui Medical University (No. YX2021‐029 (F1)).

## TRANSLATIONAL IMPACT STATEMENT

By observing the sterilizing effect of CAP, the irritation of skin of rats by CAP irradiation and the effect on the healing of scalded wounds, and the therapeutic effect of CAP on clinical acute wounds, we believe that CAP has great application prospects as a novel, economical, and safe method for treating wounds.

## Data Availability

All data generated or analyzed during this study are included in this article.

## References

[1] Wells A , Nuschke A , Yates CC . Skin tissue repair: matrix microenvironmental influences. Matrix Biol. 2016;49:25‐36. 26278492 10.1016/j.matbio.2015.08.001 PMC4753148

[2] Rahim K , Saleha S , Zhu X , Huo L , Basit A , Franco OL . Bacterial contribution in chronicity of wounds. Microb Ecol. 2017;73(3):710‐721. 27742997 10.1007/s00248-016-0867-9

[3] Eming SA , Martin P , Tomic‐Canic M . Wound repair and regeneration: mechanisms, signaling, and translation. Sci Transl Med. 2014;6(265):265sr6. 25473038 10.1126/scitranslmed.3009337 PMC4973620

[4] Sun BK , Siprashvili Z , Khavari PA . Advances in skin grafting and treatment of cutaneous wounds. Science. 2014;346(6212):941‐945. 25414301 10.1126/science.1253836

[5] Fallah N , Rasouli M , Amini MR . The current and advanced therapeutic modalities for wound healing management. J Diabetes Metab Disord. 2021;20(2):1883‐1899. 34900831 10.1007/s40200-021-00868-2 PMC8630293

[6] Bassetto F , Lancerotto L , Salmaso R , et al. Histological evolution of chronic wounds under negative pressure therapy. J Plast Reconstr Aesthet Surg. 2012;65(1):91‐99. 21885358 10.1016/j.bjps.2011.08.016

[7] Sharifi S , Hajipour MJ , Gould L , Mahmoudi M . Nanomedicine in healing chronic wounds: opportunities and challenges. Mol Pharm. 2021;18(2):550‐575. 32519875 10.1021/acs.molpharmaceut.0c00346

[8] Kim HJ , Na SW , Alodaini HA , al‐Dosary MA , Nandhakumari P , Dyona L . Prevalence of multidrug‐resistant bacteria associated with polymicrobial infections. J Infect Public Health. 2021;14(12):1864‐1869. 34801434 10.1016/j.jiph.2021.11.005

[9] Oli AN , Eze DE , Gugu TH , Ezeobi I , Maduagwu UN , Ihekwereme CP . Multi‐antibiotic resistant extended‐spectrum beta‐lactamase producing bacteria pose a challenge to the effective treatment of wound and skin infections. Pan Afr Med J. 2017;27:66. 29187917 10.11604/pamj.2017.27.66.10226 PMC5687881

[10] Haertel B , von Woedtke T , Weltmann KD , et al. Non‐thermal atmospheric‐pressure plasma possible application in wound healing. Biomol Ther. 2014;22(6):477‐490. 10.4062/biomolther.2014.105 PMC4256026 25489414

[11] Klämpfl TG , Isbary G , Shimizu T , et al. Cold atmospheric air plasma sterilization against spores and other microorganisms of clinical interest. Appl Environ Microbiol. 2012;78(15):5077‐5082. 22582068 10.1128/AEM.00583-12 PMC3416436

[12] Liu J , Yang C , Cheng C , Zhang C , Zhao J , Fu C . In vitro antimicrobial effect and mechanism of action of plasma‐activated liquid on planktonic *Neisseria gonorrhoeae* . Bioengineered. 2021;12(1):4605‐4619. 34320914 10.1080/21655979.2021.1955548 PMC8806901

[13] Maho T , Binois R , Brulé‐Morabito F , et al. Anti‐bacterial action of plasma multi‐jets in the context of chronic wound healing. Appl Sci. 2021;11(20):9598.

[14] Daeschlein G , Napp M , Lutze S , et al. Skin and wound decontamination of multidrug‐resistant bacteria by cold atmospheric plasma coagulation. J Dtsch Dermatol Ges. 2015;13(2):143‐149. 25597338 10.1111/ddg.12559

[15] Kim HY , Agrahari G , Lee MJ , Tak LJ , Ham WK , Kim TY . Low‐temperature argon plasma regulates skin moisturizing and melanogenesis‐regulating markers through yes‐associated protein. Int J Mol Sci. 2021;22(4):1895. 33672928 10.3390/ijms22041895 PMC7918577

[16] Cui HS , Cho YS , Joo SY , Mun CH , Seo CH , Kim JB . Wound healing potential of low temperature plasma in human primary epidermal keratinocytes. Tissue Eng Regen Med. 2019;16(6):585‐593. 31824821 10.1007/s13770-019-00215-w PMC6879695

[17] He R , Li Q , Shen W , et al. The efficacy and safety of cold atmospheric plasma as a novel therapy for diabetic wound in vitro and in vivo. Int Wound J. 2020;17(3):851‐863. 32168435 10.1111/iwj.13341 PMC7949340

[18] Amini MR , Sheikh Hosseini M , Fatollah S , et al. Beneficial effects of cold atmospheric plasma on inflammatory phase of diabetic foot ulcers; a randomized clinical trial. J Diabetes Metab Disord. 2020;19(2):895‐905. 33520811 10.1007/s40200-020-00577-2 PMC7843664

[19] Isbary G , Heinlin J , Shimizu T , et al. Successful and safe use of 2 min cold atmospheric argon plasma in chronic wounds: results of a randomized controlled trial. Br J Dermatol. 2012;167(2):404‐410. 22385038 10.1111/j.1365-2133.2012.10923.x PMC7161860

[20] Xu D , Wang S , Li B , et al. Effects of plasma‐activated water on skin wound healing in mice. Microorganisms. 2020;8(7):1091. 32708347 10.3390/microorganisms8071091 PMC7409103

[21] Akbiyik A , Sari D , Ercan UK , et al. The antimicrobial and tissue healing efficacy of the atmospheric pressure cold plasma on grade III infected pressure ulcer: randomized controlled in vivo experiment. J Appl Microbiol. 2021;131(2):973‐987. 33354899 10.1111/jam.14980

[22] Metelmann HR , Vu TT , Do HT , et al. Scar formation of laser skin lesions after cold atmospheric pressure plasma (CAP) treatment: a clinical long term observation. Clin Plasma Med. 2013;1(1):30‐35.

[23] Nishijima A , Fujimoto T , Hirata T , Nishijima J . Effects of cold atmospheric pressure plasma on accelerating acute wound healing: a comparative study among 4 different treatment groups. Mod Plast Surg. 2019;9(1):18‐31.

[24] Betancourt‐Ángeles M , Peña‐Eguiluz R , López‐Callejas R , et al. Treatment in the healing of burns with a cold plasma source. Int J Burns Trauma. 2017;7(7):142‐146. 29348977 PMC5768930

[25] Heinlin J , Zimmermann JL , Zeman F , et al. Randomized placebo‐controlled human pilot study of cold atmospheric argon plasma on skin graft donor sites. Wound Repair Regen. 2013;21(6):800‐807. 23937657 10.1111/wrr.12078

[26] Lou BS , Hsieh JH , Chen CM , et al. Helium/argon‐generated cold atmospheric plasma facilitates cutaneous wound healing. Front Bioeng Biotechnol. 2020;8:683. 32695763 10.3389/fbioe.2020.00683 PMC7338308

[27] Cui HS , Joo SY , Cho YS , Park JH , Kim JB , Seo CH . Effect of combining low temperature plasma, negative pressure wound therapy, and bone marrow mesenchymal stem cells on an acute skin wound healing mouse model. Int J Mol Sci. 2020;21(10):3675. 32456187 10.3390/ijms21103675 PMC7279345

[28] Duchesne C , Banzet S , Lataillade JJ , Rousseau A , Frescaline N . Cold atmospheric plasma modulates endothelial nitric oxide synthase signalling and enhances burn wound neovascularisation. J Pathol. 2019;249(3):368‐380. 31265742 10.1002/path.5323

[29] Kisch T , Helmke A , Schleusser S , et al. Improvement of cutaneous microcirculation by cold atmospheric plasma (CAP): results of a controlled, prospective cohort study. Microvasc Res. 2016;104:55‐62. 26655582 10.1016/j.mvr.2015.12.002

[30] Schmidt A , Liebelt G , Nießner F , von Woedtke T , Bekeschus S . Gas plasma‐spurred wound healing is accompanied by regulation of focal adhesion, matrix remodeling, and tissue oxygenation. Redox Biol. 2021;38:101809. 33271456 10.1016/j.redox.2020.101809 PMC7710641

[31] Keidar M , Walk R , Shashurin A , et al. Cold plasma selectivity and the possibility of a paradigm shift in cancer therapy. Br J Cancer. 2011;105(9):1295‐1301. 21979421 10.1038/bjc.2011.386 PMC3241555

[32] Vaquero J , Judée F , Vallette M , et al. Cold‐atmospheric plasma induces tumor cell death in preclinical in vivo and in vitro models of human cholangiocarcinoma. Cancer. 2020;12(5):1280. 10.3390/cancers12051280 PMC7281400 32438553

[33] Vandamme M , Robert E , Pesnel S , et al. Antitumor effect of plasma treatment on U87 glioma xenografts: preliminary results. Plasma Process Polym. 2010;7(3–4):264‐273.

[34] Rasouli M , Mehdian H , Hajisharifi K , Amini E , Ostrikov K(K) , Robert E . Plasma‐activated medium induces apoptosis in chemotherapy‐resistant ovarian cancer cells: high selectivity and synergy with carboplatin. Plasma Process Polym. 2021;18(9):2100074.

[35] Daeschlein G , Arnold A , Lutze S , et al. Treatment of recalcitrant actinic keratosis (AK) of the scalp by cold atmospheric plasma. Cogent Med. 2017;4(1):1412903.

[36] Kim YJ , Lim DJ , Lee MY , Lee WJ , Chang SE , Won CH . Prospective, comparative clinical pilot study of cold atmospheric plasma device in the treatment of atopic dermatitis. Sci Rep. 2021;11(1):14461. 34262113 10.1038/s41598-021-93941-y PMC8280139

[37] Heinlin J , Isbary G , Stolz W , et al. A randomized two‐sided placebo‐controlled study on the efficacy and safety of atmospheric non‐thermal argon plasma for pruritus. J Eur Acad Dermatol Venereol. 2013;27(3):324‐331. 22188329 10.1111/j.1468-3083.2011.04395.x

[38] Xiong Z , Roe J , Grammer TC , Graves DB . Plasma treatment of onychomycosis. Plasma Process Polym. 2016;13(6):588‐597.

[39] Gan L , Duan J , Zhang S , et al. Cold atmospheric plasma ameliorates imiquimod‐induced psoriasiform dermatitis in mice by mediating antiproliferative effects. Free Radic Res. 2019;53(3):269‐280. 30663913 10.1080/10715762.2018.1564920

[40] Chutsirimongkol C , Boonyawan D , Polnikorn N , Techawatthanawisan W , Kundilokchai T . Non‐thermal plasma for acne and aesthetic skin improvement. Plasma Med. 2014;4:1‐4.

[41] Bolouki N , Hsu YN , Hsiao YC , et al. Cold atmospheric plasma physically reinforced substances of platelets‐laden photothermal‐responsive methylcellulose complex restores burn wounds. Int J Biol Macromol. 2021;192:506‐515. 34599990 10.1016/j.ijbiomac.2021.09.168

[42] Yang Y , Guo J , Zhou X , et al. A novel cold atmospheric pressure air plasma jet for peri‐implantitis treatment: an in vitro study. Dent Mater J. 2018;37(1):157‐166. 29176301 10.4012/dmj.2017-030

[43] Yu F . Effect of a low temperature plasma knife on the treatment of chronic tonsillitis and its effect on T lymphocyte subsets. Am J Transl Res. 2021;13(4):2447‐2455. 34017403 PMC8129394

[44] Lee HY , Lee HJ , Kim GC , Choi JH , Hong JW . Plasma cupping induces VEGF expression in skin cells through nitric oxide‐mediated activation of hypoxia inducible factor 1. Sci Rep. 2019;9(1):3821. 30846730 10.1038/s41598-019-40086-8 PMC6405951

[45] Rice LB . Federal funding for the study of antimicrobial resistance in nosocomial pathogens: no ESKAPE. J Infect Dis. 2008;197(8):1079‐1081. 18419525 10.1086/533452

[46] Friedman ND , Temkin E , Carmeli Y . The negative impact of antibiotic resistance. Clin Microbiol Infect. 2016;22(5):416‐422. 26706614 10.1016/j.cmi.2015.12.002

[47] Alkawareek MY , Algwari QT , Laverty G , et al. Eradication of *Pseudomonas aeruginosa* biofilms by atmospheric pressure non‐thermal plasma. PLoS One. 2012;7(8):e44289. 22952948 10.1371/journal.pone.0044289 PMC3432087

[48] Yin W , Wang Y , Liu L , He J . Biofilms: the microbial “protective clothing” in extreme environments. Int J Mol Sci. 2019;20(14):3423. 31336824 10.3390/ijms20143423 PMC6679078

[49] Hall CW , Mah TF . Molecular mechanisms of biofilm‐based antibiotic resistance and tolerance in pathogenic bacteria. FEMS Microbiol Rev. 2017;41(3):276‐301. 28369412 10.1093/femsre/fux010

[50] Singh NP , Rani M , Gupta K , Sagar T , Kaur IR . Changing trends in antimicrobial susceptibility pattern of bacterial isolates in a burn unit. Burns. 2017;43(5):1083‐1087. 28153582 10.1016/j.burns.2017.01.016

[51] Percival SL , Emanuel C , Cutting KF , Williams DW . Microbiology of the skin and the role of biofilms in infection. Int Wound J. 2012;9(1):14‐32. 21973162 10.1111/j.1742-481X.2011.00836.x PMC7950481

[52] Flynn PB , Higginbotham S , Nid'a HA , et al. Bactericidal efficacy of atmospheric pressure non‐thermal plasma (APNTP) against the ESKAPE pathogens. Int J Antimicrob Agents. 2015;46(1):101‐107. 25963338 10.1016/j.ijantimicag.2015.02.026

[53] Shen W , Xu W , Chen H . Immunological mechanisms of scarring and their psychological impact on patients. Am J Clin Exp Immunol. 2021;10(3):65‐70. 34824895 PMC8610804

[54] Barrett LW , Fear VS , Waithman JC , Wood FM , Fear MW . Understanding acute burn injury as a chronic disease. Burns Trauma. 2019;7:23. 31534977 10.1186/s41038-019-0163-2 PMC6745803

[55] Hartwig S , Preissner S , Voss JO , et al. The feasibility of cold atmospheric plasma in the treatment of complicated wounds in cranio‐maxillo‐facial surgery. J Craniomaxillofac Surg. 2017;45(10):1724‐1730. 28843407 10.1016/j.jcms.2017.07.008

[56] Troncoso F , Herlitz K , Acurio J , et al. Advantages in wound healing process in female mice require upregulation A_2A_‐mediated angiogenesis under the stimulation of 17β‐estradiol. Int J Mol Sci. 2020;21(19):7145. 32998232 10.3390/ijms21197145 PMC7583763

[57] Routley CE , Ashcroft GS . Effect of estrogen and progesterone on macrophage activation during wound healing. Wound Repair Regen. 2009;17(1):42‐50. 19152650 10.1111/j.1524-475X.2008.00440.x

[58] Gilliver SC , Ruckshanthi JP , Atkinson SJ , et al. Androgens influence expression of matrix proteins and proteolytic factors during cutaneous wound healing. Lab Investig. 2007;87(9):871‐881. 17607299 10.1038/labinvest.3700627

[59] Gilliver SC , Ruckshanthi JP , Hardman MJ , et al. Sex dimorphism in wound healing: the roles of sex steroids and macrophage migration inhibitory factor. Endocrinology. 2008;149(11):5747‐5757. 18653719 10.1210/en.2008-0355

[60] Maisch T , Bosserhoff AK , Unger P , et al. Investigation of toxicity and mutagenicity of cold atmospheric argon plasma. Environ Mol Mutagen. 2017;58(3):172‐177. 28370324 10.1002/em.22086

[61] Schmidt A , von Woedtke T , Stenzel J , et al. One year follow‐up risk assessment in SKH‐1 mice and wounds treated with an argon plasma jet. Int J Mol Sci. 2017;18(4):868. 28422070 10.3390/ijms18040868 PMC5412449

[62] Evert K , Kocher T , Schindler A , et al. Repeated exposure of the oral mucosa over 12 months with cold plasma is not carcinogenic in mice. Sci Rep. 2021;11(1):20672. 34667240 10.1038/s41598-021-99924-3 PMC8526716

[63] Stancampiano A , Chung TH , Dozias S , Pouvesle JM , Mir LM , Robert E . Mimicking of human body electrical characteristic for easier translation of plasma biomedical studies to clinical applications. IEEE Trans Radiat Plasma Med Sci. 2019;4(3):335‐342.

